# Identification of stable reference genes in peripheral blood mononuclear cells from type 2 diabetes mellitus patients

**DOI:** 10.1038/s41598-023-27460-3

**Published:** 2023-01-10

**Authors:** Ankita Hazarika, Bajanai Nongkhlaw, Arpita Mukhopadhyay

**Affiliations:** 1 Division of Nutrition, St. John’s Research Institute, St. John’s National Academy of Health Sciences, Sarjapur Road, Bangalore, India; 2 Present Address: Department of Pathology, North Eastern Indira Gandhi Regional Institute of Health and Medical Sciences, Shillong, Meghalaya India

**Keywords:** Type 2 diabetes, Transcriptomics

## Abstract

Reference genes are obligatory for accurate normalization of mRNA transcript levels across samples and experimental conditions in Real Time-polymerase chain reaction (qRT-PCR) based quantitative gene expression assays. Selection of stably expressed reference genes is therefore crucial for ensuring reproducibility of such assays. However, there is a complete dearth of data on stability of commonly used reference genes in Peripheral Blood Mononuclear Cells (PBMCs) from Type 2 diabetes mellitus (T2DM) patients. We have evaluated the gene expression stability of 4 widely used reference genes (Beta-actin, *ACTB*; Peptidylprolyl Isomerase B, *PPIB*; Tyrosine 3 Monooxygenase/Tryptophan 5-Monooxygenase Activation Protein Zeta, *YWHAZ*; and Glyceraldehyde-3-Phosphate Dehydrogenase, *GAPDH*); in PBMCs from 39 T2DM patients and 47 normoglycemic (NGT) subjects. *ACTB* and *YWHAZ* were found to be the most stable genes in PBMCs from T2DM patients and therefore, can be recommended as suitable reference genes in similar contexts. *GAPDH* and *PPIB* expressions were not stable in PBMCs from T2DM patients. On using *ACTB* and *YWHAZ* as reference genes for measuring relative expression of *GAPDH* and *PPIB* in these subjects, relative *GAPDH* expression was found to be significantly lower in female T2DM patients, compared to female NGT subjects [*GAPDH* relative normalization unit (RNU): female T2DM (n = 19), median (Q1, Q3): 9.0 (8.1, 9.9); female NGT (n = 18): median (Q1, Q3): 10.1 (9.1, 11.0); *P* = 0.034]. Dysregulation of *GAPDH* in PBMCs from female T2DM patients could be associated with sex-specific differences in pathogenesis and outcomes of T2DM.

## Introduction

Type 2 diabetes mellitus (T2DM) is one of the top ten causes of death worldwide^1^. The immune system plays a vital role in the pathogenesis of T2DM, both at a causal level and during progression of the disease^2–6^. This has implications in terms of increased risk of infections in diabetics^7^. Peripheral blood mononuclear cells (PBMCs) fight infection as a critical part of the immune system^8^ and are easily sourced and therefore, widely used representatives of the immune system from diabetics. Further, they have the potential to represent relevant features of the body's immunometabolic condition^9,10^. Measurement of expression of genes in PBMCs is a well-used method of deciphering the effect of drugs^11,12^ or of intervention regimes in randomized, controlled trials^13^ on the diabetic immune system. Several studies have used quantitative Real Time-polymerase chain reaction (qRT-PCR) based assays to compare transcript abundance in PBMCs of healthy vs T2DM patients^14–16^. qRT-PCR is a strong technology for detecting changes in gene expression and is regarded as the “gold standard” in the field of mRNA quantification^17^. However, to assure robustness and repeatability of the resultant data, this method necessitates a normalization strategy when gene expression is measured in ‘relative’ and not ‘absolute’ terms^18,19^. Normalization of the expression data is achieved by accounting for the variability introduced due to amount of the PCR template (complimentary DNA, cDNA, generated from mRNA transcripts, in case of gene expression assays) at the beginning of the PCR assay across the samples through use of reference or housekeeping genes as internal controls^20^.

A stable reference gene is expected to not show inter-subject variation or be affected by the disease condition^21,22^. Glyceraldehyde-3-Phosphate Dehydrogenase (*GAPDH*), is extensively used as a reference gene for measuring relative gene expression in PBMCs from diabetics^23–25^. Nevertheless, multiple studies have reported that hyperglycaemia can alter *GAPDH* expression in cultured cells and animal models^26–28^.

Meticulous selection of reference genes is of utmost importance for accurate analysis of gene expression data. Interestingly, even though PBMCs from diabetics have been extensively used for immune-associated gene expression studies, only a single study has evaluated stability of reference genes in PBMCs from type 1 diabetics exist^22^. There is a complete dearth of similar studies on T2DM patients. In this backdrop, the aim of this study was to evaluate stability of expression of 4 widely used reference genes: Beta-actin (*ACTB*), Peptidylprolyl Isomerase B (*PPIB*), Tyrosine 3-Monooxygenase/Tryptophan 5-Monooxygenase Activation Protein Zeta (*YWHAZ*), and *GAPDH* in PBMCs from T2DM patients and healthy control subjects. The primary reason for the selection of these 4 genes in the study was that they have been widely used as reference genes in gene-expression studies on human PBMCs—*GAPDH*^11,25^, *ACTB*^11,29^, *PPIB*^29^, *YWHAZ*^30,31^. Further, the 4 genes chosen for this study have different physiological and cellular roles: cytoskeleton (*ACTB*)^32^, carbohydrate metabolism (*GAPDH*)^33^, protein folding (*PPIB*)^34^, an adapter protein in signaling pathways (*YWHAZ*)^35^. Therefore, the probability of the diabetic condition in a subset of the study subjects affecting expression of all 4 of them was likely minimal. To the best of our knowledge, this is the first study to investigate the appropriateness of these 4 potential reference genes in PBMCs, including a subgroup analysis based on sex of the subjects.

## Results

### Anthropometric and clinical characteristics of the study subjects

The anthropometric and clinical characteristics of the 86 study subjects are summarized in Table 1. Weight, height, BMI, waist, hip, Waist:Hip ratio, and blood pressure were similar between the NGT and T2DM groups. The T2DM group was older with a mean age of 47.6 ± 8.3 years (*p * ˂  0.001). HbA1c (8.7 ± 1.7, *p* ˂ 0.001), fasting glucose (183.3 ± 79, *p* ˂ 0.001), and triglyceride (203.9 ± 99.6 mg/dL, *p* ˂ 0.001) levels were higher in T2DM patients.

**Table 1:** Subject characteristics and metabolic profile of the 86 study subjects. Data presented as mean ± SD.

	All samples (n = 86)	NGT (n = 47)	T2DM (n = 39)	*P*
Gender (M/F)^#^	49/37	29/18	20/19	0.331
Age (years)	42.4 ± 8.8	38.1 ± 6.7	47.6 ± 8.3	** ˂ 0.001**
Height (cm)	161.3 ± 9.5	163.3 ± 9.9	158.8 ± 8.5	**0.028**
Weight (kg)	65.5 ± 9.6	67.1 ± 10	63.7 ± 8.8	0.099
BMI (kg/m^2^)	25.1 ± 2.4	25.1 ± 2.4	25.2 ± 2.5	0.819
Waist-hip ratio (cm)	0.9 ± 0.1	0.9 ± 0.1	0.9 ± 0.1	0.405
Systolic blood pressure (mmHg)	120.0 ± 15.0	119.0 ± 14.0	121.0 ± 16.0	0.404
Diastolic blood pressure (mmHg)	80.0 ± 8.0	80.0 ± 9.0	80.0 ± 7.0	0.969
Body fat (%)	35.2 ± 7.5	34.9 ± 6.9	35.5 ± 8.4	0.710
Fat mass (kg)	22.1 ± 5.2	22.3 ± 4.6	21.8 ± 5.9	0.681
Android fat (%)	43.7 ± 6.5	43.7 ± 6.3	43.8 ± 6.7	0.947
Gynoid fat (%)	39.6 ± 9.0	40.1 ± 8.2	39.0 ± 9.9	0.561
Android: gynoid ratio	1.1 ± 0.2	1.1 ± 0.2	1.2 ± 0.2	0.250
Appendicular lean mass (kg)	41.0 ± 8.1	42.2 ± 8.6	39.5 ± 7.3	0.125
Bone Mineral content (kg)	2.5 ± 0.5	2.6 ± 0.5	2.4 ± 0.4	**0.019**

Metabolic profile^	All samples (n = 86)	NGT (n = 47)	T2DM (n = 39)	
Gender (M/F)	49/37	29/18	20/19	
HbA1c (%)	6.8 ± 2.1	5.3 ± 0.3	8.7 ± 1.7	** ˂ 0.001**
Fasting glucose (mg/dL)	129.4 ± 72.6	84.7 ± 9.3	183.3 ± 79	** ˂ 0.001**
Fasting Insulin (mU/L)	11.7 ± 7.5	10.6 ± 7.1	13.0 ± 7.8	0.132
C-Peptide (ng/mL)	3.0 ± 1.5	2.5 ± 1.1	3.6 ± 1.7	**0.001**
Cholesterol (mg/dL)	192.5 ± 41.4	178.5 ± 35.9	209.4 ± 41.7	**0.001**
HDL cholesterol (mg/dL)	42.8 ± 9.0	42.9 ± 8.3	42.7 ± 9.8	0.930
LDL cholesterol (mg/dL)	123.2 ± 29.6	118 ± 27.5	129.6 ± 31.1	0.073
Triglycerides (mg/dL)	157.1 ± 88.0	118.2 ± 52.2	203.9 ± 99.6	** ˂ 0.001**
Creatinine (mg/dL)	0.8 ± 0.2	0.8 ± 0.2	0.8 ± 0.3	0.639

^Blood was drawn from fasting volunteers. *P* values are from the independent t-test. *P* ˂ 0.05 is shown in bold. ^#^*P* value from Chi-square test.

### Expression profiling of the 4 reference genes

To identify the most and the least stable reference gene in PBMCs from T2DM patients, we tested gene-expression stability of 4 potential reference genes (*ACTB, YWHAZ, PPIB*, and *GAPDH*) in PBMCs of T2DM patients (n = 39) and compared them to that of NGT subjects (n = 47). These genes were selected as they have been widely used as reference genes for gene-expression studies in PBMCs from T2DM patients^14–16^. The raw Ct values for the 4 potential reference genes for the total 86 study subjects are provided in Supplementary Table 1. The raw Ct values were not different between the NGT and T2DM groups for any of the potential reference genes evaluated (Table 2).

**Table 2:** Descriptive statistics of raw C_t_ values of the 4 reference genes (*ACTB, PPIB, YWHAZ* and *GAPDH*) in PBMCs of the 86 study subjects categorized into normoglycemic controls (NGT, n = 47) and Type 2 diabetic patients (T2DM, n = 39).

Reference genes		All samples (n = 86)	NGT (n = 47)	T2DM (n = 39)	*P*
*ACTB*	Mean ± SD	21.3 ± 2.6	21.0 ± 2.7	21.6 ± 2.5	0.264
	Median (Q1, Q3)	21.3 (18.9, 23.3)	20.4 (18.8, 22.9)	21.7 (20.0, 23.5)	
	Range (Min, Max)	9.6 (16.9, 26.4)	9.6 (16.9, 26.4)	8.9 (17.1, 25.9)	
*GAPDH*	Mean ± SD	30.0 ± 4.2	29.5 ± 4.1	30.5 ± 4.4	0.315
	Median (Q1, Q3)	29.9 (26.7, 32.0)	29.5 (25.8, 32.3)	30.4 (27.4, 32)	
	Range (Min, Max)	17.1 (22.9, 40.0)	17.1 (22.9, 40.0)	17.0 (23, 40.0)	
*YWHAZ*	Mean ± SD	26.5 ± 3.6	26.2 ± 3.5	26.8 ± 3.6	0.530
	Median (Q1, Q3)	26.1 (23.6, 28.5)	25.6 (23.6, 28.5)	27.3 (24.2, 28.5)	
	Range (Min, Max)	19.6 (20.4, 40.0)	18.6 (21.4, 40.0)	19.6 (20.4, 40.0)	
*PPIB*	Mean ± SD	30.2 ± 4.6	30.1 ± 5.1	30.4 ± 3.8	0.190
	Median (Q1, Q3)	29.5 (26.7, 32.6)	29.0 (26.3, 32.2)	30.9 (27.6, 32.9)	
	Range (Min, Max)	17.4 (22.6, 40.0)	16.5 (23.5, 40.0)	17.4 (22.6, 40.0)	

*P* values are from Mann Whitney Test.

### Expression stability analysis of the reference genes

**Figure 1:**
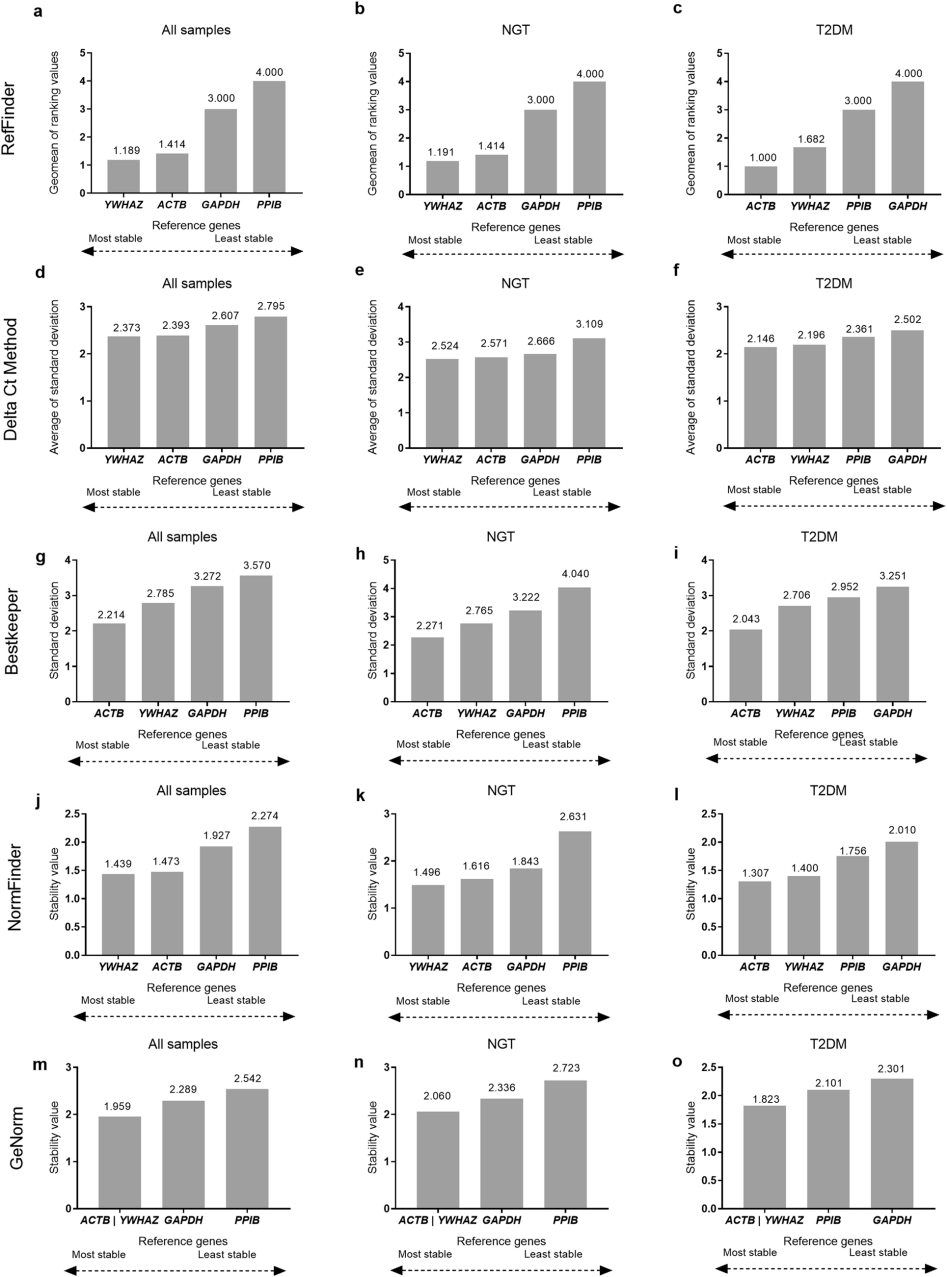
Expression stability analysis and ranking of the 4 reference genes provided by RefFinder tool and different algorithms considered by RefFinder for of all subjects (n = 86), NGT group (n = 47) and T2DM group (n = 39). Comprehensive ranking of the reference genes provided by RefFinder for (**a**) All samples, (**b**) NGT group and (**c**) T2DM group. Gene expression stability plots and subsequent ranking of the reference genes by Delta Ct method^41^ for (**d**) All samples**, **(**e**) NGT and (**f**) T2DM; BestKeeper^40^ for (**g**) All samples, (**h**) NGT and (**i**) T2DM; NormFinder^39^ for (**j**) All samples, (**k**) NGT and (**l**) T2DM; and GeNorm^37,38^, based on average stability value (M value) for (**m**) All samples, (**n**) NGT and (**o**) T2DM. The most stable genes are on the left side and the least stable genes are arranged on the right side. The stability values, for each gene are indicated as data labels on the bar-graphs.

To identify the stability of expression of the 4 potential reference genes (*ACTB, PPIB, YWHAZ*, and *GAPDH*) in PBMCs from all study samples and separately in NGT and T2DM groups, the raw Ct values were assessed using the RefFinder tool^36^. The raw Ct values were directly imported into the RefFinder tool, which takes into account the stability rankings generated by four algorithms: GeNORM^37,38^, NormFinder^39^, BestKeeper^40^, and delta Ct^41^ to provide a comprehensive stability ranking to the potential reference genes^36,42^. The stability of the 4 potential reference genes, examined using the RefFinder tool, for all samples (n = 86), NGT (n = 47), and T2DM (n = 39) groups is depicted in Fig. 1. Across all 4 algorithms used and the comprehensive ranking analysis by the RefFinder tool, *ACTB* and/or *YWHAZ* were predicted to be the most stable genes, while *PPIB* was consistently the least stable gene in all samples and separately in the NGT group and *GAPDH* was the least stable gene in the T2DM group (Fig. 1).

The comprehensive ranks of gene stability were obtained from RefFinder which assigns an appropriate weight to each gene to calculate the geometric mean from stability values obtained in the 4 algorithms as mentioned above and provides an overall final stability ranking to the genes. The most stable gene in all samples was *YWHAZ* while *PPIB* was the least stable gene (Fig. 1a). The most and least stable genes in the NGT group were *YWHAZ* and *PPIB* respectively (Fig. 1b); while in the T2DM group, the most and least stable gene were *ACTB* and *GAPDH* respectively (Fig. 1c).

In the ΔCt method, a comparison of the relative expression of pairs of genes is done for each sample, and ranks for stability are provided in accordance with the repeatability in the difference in expression across the samples^41^. Based on this approach, *YWHAZ* and *PPIB* were the most and least stable genes respectively in all samples and NGT group, and *ACTB* and *GAPDH* were the most and least stable genes respectively in the T2DM group (Fig. 1d, e, f).

The Bestkeeper approach determines the stability of genes based on the principle that the expression of ideal reference genes is highly correlated. The program constructs a correlative index out of it to determine the stability values (standard deviation) of the reference genes^40^. *ACTB* was predicted to be the most stable reference gene in all samples, NGT, and T2DM groups. *PPIB* was the least stable gene for all samples and the NGT group (Fig. 1g, h). *GAPDH* was the least stable gene in the T2DM group (Fig. 1i).

NormFinder is based on the consideration that any reference genes would show some extent of variation in their expression across the sample set. It predicts the stability measures based on the overall variation^39^. NormFinder predicted *YWHAZ* to be the most stable gene in the all samples and the NGT group (Fig. 1j, 1k) while *ACTB* was the most stable gene in the T2DM group (Fig. 1l). *PPIB* was the least stable gene for all samples and the NGT group (Fig. 1j, k). *GAPDH* was the least stable gene in the T2DM group (Fig. 1l).

The stability of reference genes in GeNorm is based on the principle that the ratio of expression between two ideal reference genes should remain the same under all experimental conditions. The algorithm measures the stability of a reference gene considering that any deviation from the expression ratio will reflect an inconsistency in the expression of either of the reference genes and any increase in this ratio indicates a corresponding decrease in gene stability. GeNorm assigns a gene stability value M to all the reference genes, which is the average pairwise variation of a gene with all other reference genes included in the study. The most stable genes are the ones with the least M values^37^. For all samples, NGT, and T2DM groups, the most stable genes were predicted to be *ACTB/YWHAZ* (Fig. 1m, n, o).

### Expression stability analysis of the reference genes separately in males and females

**Figure 2:**
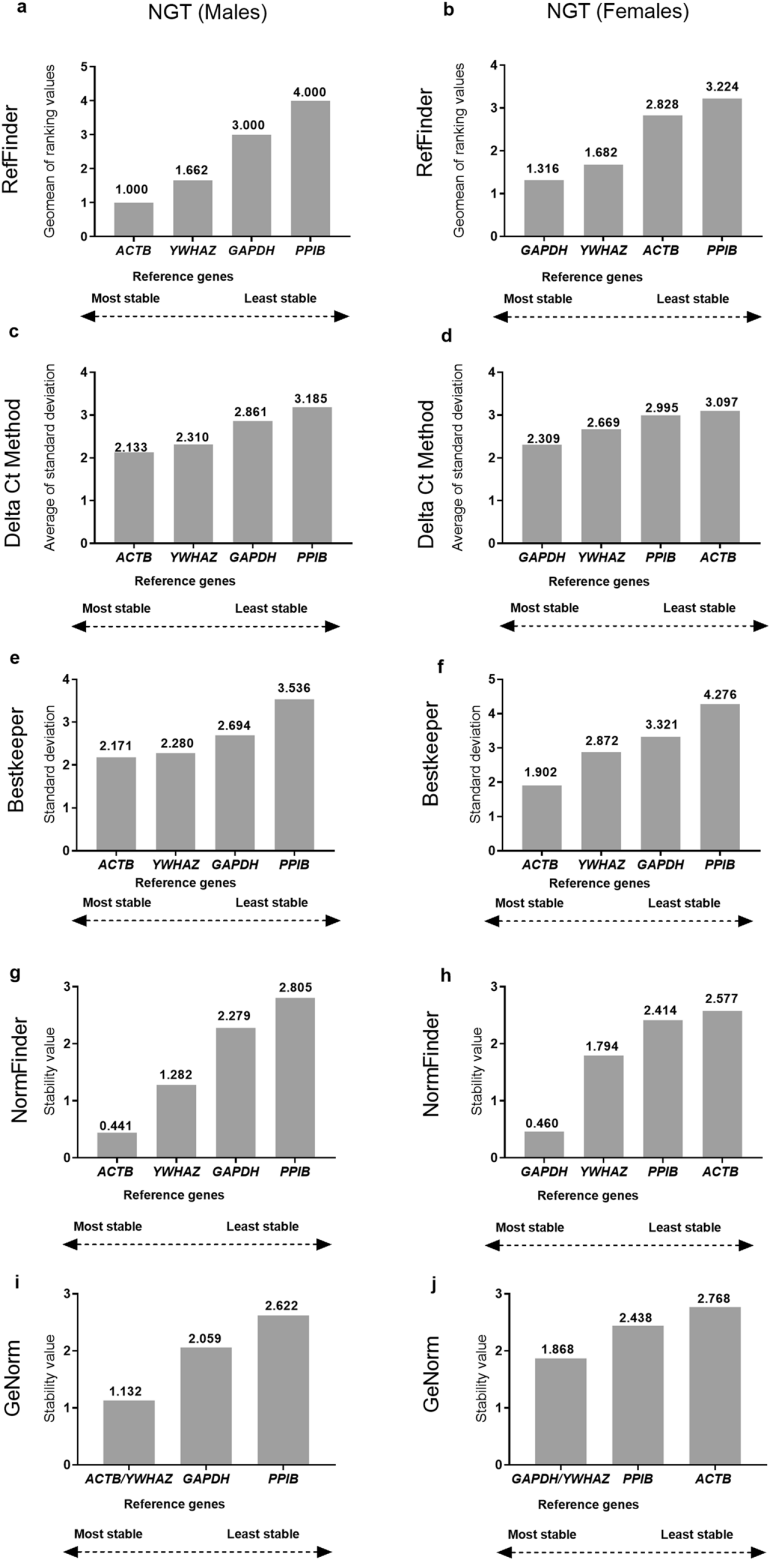
Gender-based expression stability analysis and ranking of the 4 reference genes provided by RefFinder tool and different algorithms considered by RefFinder for the NGT group (n = 47) and separately for NGT Males (n = 29) and NGT Females (n = 18). Comprehensive ranking of the reference genes provided by RefFinder for (**a**) Males, (**b**) Females; ranking of the reference genes by Delta Ct method^41^ for (**c**) Males, (**d**) Females; BestKeeper^40^ for (**e**) Males, (**f**) Females; NormFinder^39^ for (**g**) Males, (**h**) Females; and GeNorm^37,38^, based on average stability value (M value) for (**i**) Males, (**j**) Females; The most stable genes are on the left side and the least stable genes are arranged on the right side. The stability values, for each gene are indicated as data labels on the bar-graphs.

We have additionally assessed the stability of the 4 potential reference genes separately in males (NGT: n = 29, T2DM: n = 20) and females (NGT: n = 18, T2DM: n = 19) within the NGT and T2DM groups. Within NGT males, *ACTB* and *PPIB* were consistently predicted as the most and least stable genes, respectively (Fig. 2a, c, e, g, i). For NGT females, there was no consistency in the prediction of most stable and least stable genes. However, based on comprehensive ranking by the RefFinder tool, *PPIB* was found to be the least stable gene in both males and females of the NGT group (Fig. 2a, b).

**Figure 3:**
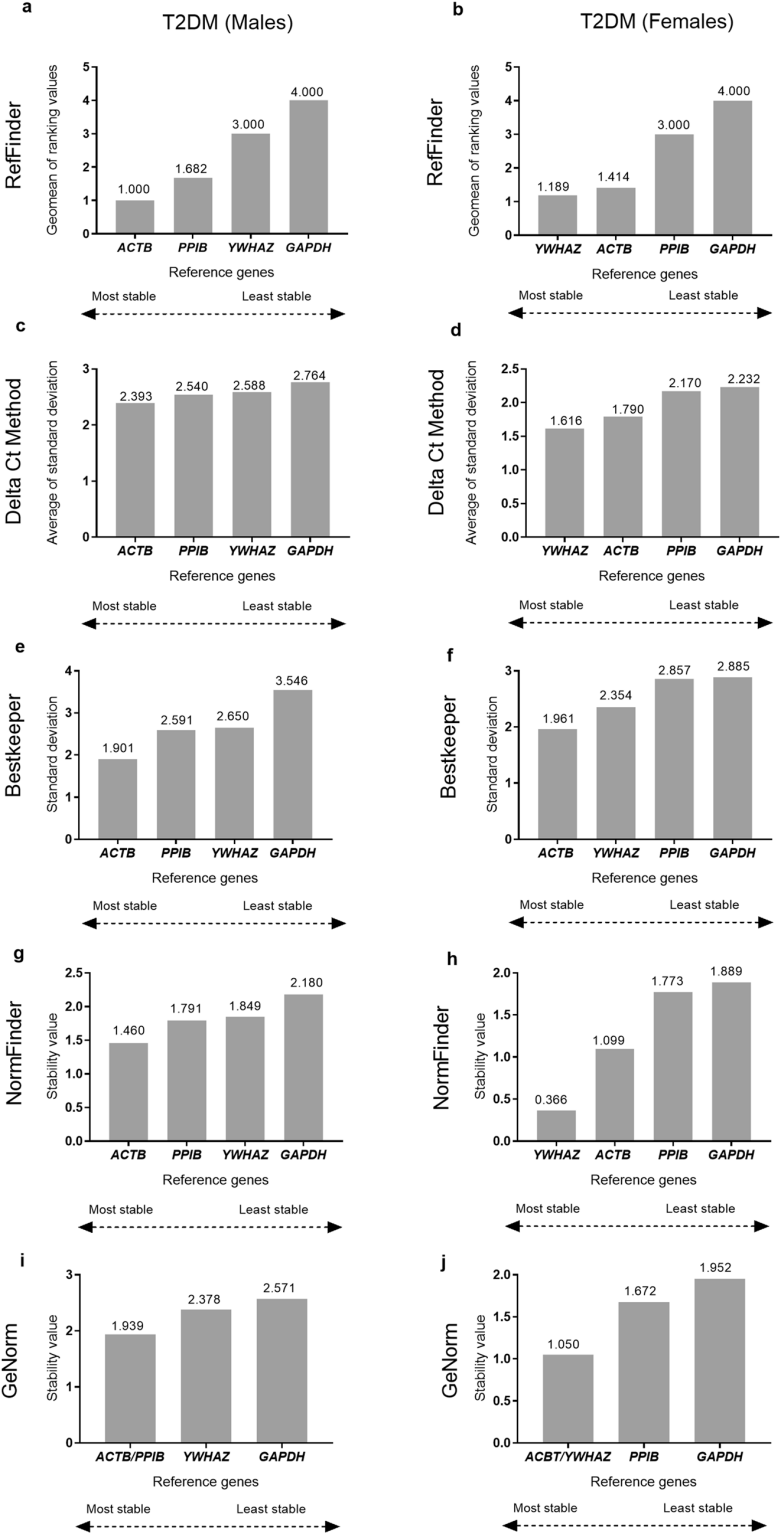
Gender-based expression stability analysis and ranking of the 4 reference genes provided by RefFinder tool and different algorithms considered by RefFinder for the T2DM group (n = 39) and separately for T2DM Males (n = 20) and T2DM Females (n = 19). Comprehensive ranking of the reference genes provided by RefFinder for (**a**) Males, (**b**) Females; Gene expression stability plots and subsequent ranking of the reference genes by Delta Ct method^41^ for (**c**) Males, (**d**) Females; BestKeeper^40^ for (**e**) Males, (**f**) Females; NormFinder^39^ for (**g**) Males, (**h**) Females; and GeNorm^37,38^, based on average stability value (M value) for (**i**) Males, (**j**) Females; The most stable genes are on the left side and the least stable genes are arranged on the right side. The stability values, for each gene are indicated as data labels on the bar-graphs.

The four algorithms, delta Ct, BestKeeper, NormFinder, and GeNorm, as well as the RefFinder tool's comprehensive ranking, consistently predicted *GAPDH* to be the least stable gene in both males and females with T2DM subjects (Fig. 3). *ACTB* was predicted to be the most stable gene in T2DM males (Fig. 3a, c, e, g, i), while *YWHAZ* was predicted to be the most stable gene in T2DM females by all algorithms except Bestkeeper (Fig. 3b, d, h, j).

### Relative gene expression analysis of *GAPDH* and *PPIB*

**Figure 4:**
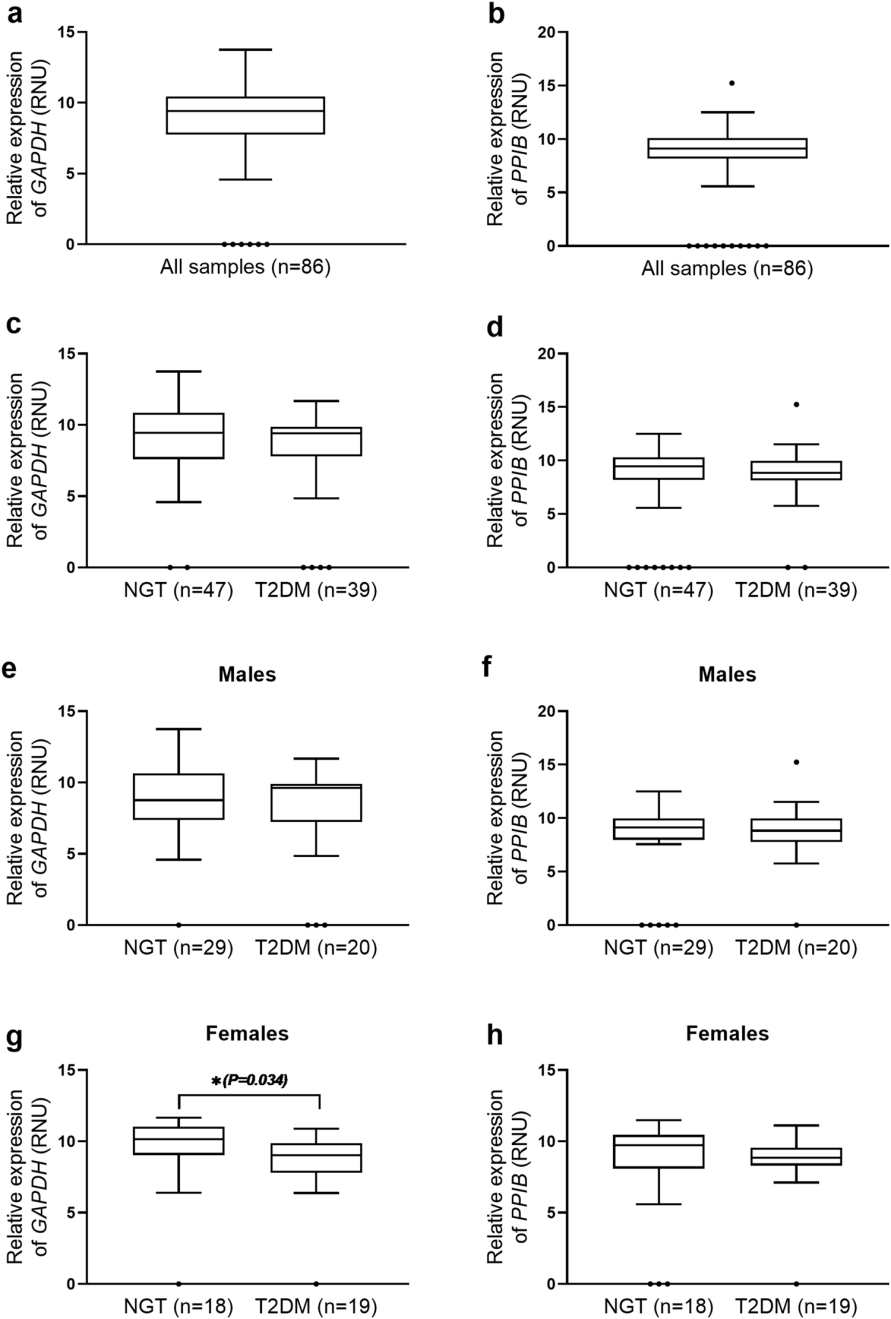
Boxplots of relative expression of *GAPDH* and *PPIB* expressed as relative normalization units (RNU) with *ACTB* and *YWHAZ* as reference genes, in all study samples (n = 86); NGT group (n = 47); T2DM group (n = 39); male NGT (n = 29) and T2DM (n = 20) subjects and female NGT (n = 18) and T2DM (n = 19) subjects. Relative expression of *GAPDH* in all study samples (**a**)**,** NGT and T2DM groups (**c**)**,** in male NGT and T2DM subjects (**e**) and in female NGT and T2DM subjects (**g**). Relative expression of *PPIB* in all samples (**b**), in NGT and T2DM groups (**d**)**,** in male NGT and T2DM subjects (**f**) and in female NGT and T2DM subjects (**h**). Outliers are represented as dots “(black shaded circle)”. The median values are represented as lines across the box. The lower and the upper boxes represent the first and third quartile, respectively. Whiskers represent the maximum and minimum value. *P* values are from Mann Whitney test. Calculation of relative expression for *GAPDH* and *PPIB* as target genes was done based on a modification of the ΔCt method using *ACTB* and *YWHAZ* as reference genes; [ΔCt = Ct_(target gene)_—Ct_(Reference genes mean)_]. RNU was calculated as, RNU = 15−ΔCt^43^.

As *ACTB* and *YWHAZ* were consistently predicted to be the most stable genes and *GAPDH* and *PPIB* the least stable ones in our study samples, we next assessed relative transcript abundances of *GAPDH* and *PPIB* in our study samples using *ACTB* and *YWHAZ* as the reference genes (Fig. 4 and Supplementary Table 2). Relative expression of *PPIB* in NGT and T2DM groups, either overall, or separately within males and within females, was similar (Fig. 4d, f, h). Relative expression of *GAPDH* in NGT and T2DM groups, overall, and within males was similar (Fig. 4c, e). The relative expression of *GAPDH* was significantly lower in T2DM females compared to that in NGT females (*P* = 0.034) (Fig. 4g).

## Discussion

Progression of T2DM and altered immunity go hand in hand^2^ with both innate^3^ and adaptive^4^ branches of the immune system implicated in causation and pathophysiology of T2DM, including at the level of developmental programming of T2DM in growth-restricted births^44^, leading to clinically relevant implications for increased susceptibility and worse progression of infections, including that of COVID-19^7^. Against this backdrop, measuring gene expression changes in the PBMCs, easily sourced immune cells from human T2DM subjects, using the rapid, highly accurate and inexpensive real-time qRT-PCR technique^45^ forms the bedrock of advancing our understanding of the altered immune system in T2DM. However, reproducibility of qRT-PCR based relative gene expression measurements depends on choosing reference genes, whose expression remain stable across individual, tissue type, morbidity conditions and any other relevant experimental parameters^10^.

In the current study, we have evaluated the stability of 4 commonly used reference genes: *ACTB, PPIB, YWHAZ* and *GAPDH* in PBMCs from 86 subjects: T2DM (n = 39) and NGT (n = 47). *ACTB* and *YWHAZ* were the most stable reference genes in our study samples. As such, we recommend using *ACTB* and *YWHAZ* as reference genes when measuring relative gene expression in PBMCs in settings similar to ours.

*PPIB* was the least stable reference gene in PBMC samples from NGT subjects in our study. *PPIB* is a member of the Peptidyl-prolyl cis–trans isomerase (PPIase) family which catalyses the *cis*–trans isomerization of proline imidic peptide bonds and is essential for protein folding during protein synthesis in the cell^46^. We could not find any earlier studies evaluating the stability of *PPIB* expression in PBMCs from T2DM patients. Further, in contrast to our findings, Pachot et al. have recommended *PPIB* as a suitable reference gene for quantification of gene expression in peripheral whole blood^47^. However, it needs to be noted here that unlike our study, Pachot et al. had conducted measurement of *PPIB* by qRT-PCR in 91 whole blood samples from a combined group of inflammatory patients and healthy volunteers, without conducting further sub-group analysis on stability of expression *PPIB*, grouping the subjects based on either their inflammatory status or sex.

*GAPDH* was the least stable gene in PBMCs from T2DM patients in our study, both overall and separately in T2DM males and females. Similar to our findings, Kar et al. reported high variance in *GAPDH* expression in PBMCs from type 1 diabetics^48^. These findings are in line with prior research in animal models and cell lines on the effect of hyperglycaemia on *GAPDH* expression. Du et al. reported that in the aortae of wild-type mice and bovine aortic endothelial cells, hyperglycaemia-induced overproduction of superoxide decreased GAPDH activity^28,49^. The feed-back impact of hyperglycaemia on glucose sensitive elements in this gene might explain why *GAPDH* is less stable in diabetic environments^28,50^.

As *GAPDH* and *PPIB* were the least stable genes in our study samples, we further assessed relative expression of *GAPDH* and *PPIB* using *ACTB* and *YWHAZ* as reference genes. Unique to our study, we observed significant reduction of relative expression of *GAPDH* in PBMCs from T2DM females compared to that from NGT females while no difference was observed between T2DM and NGT males. Our findings are supported by those of Grindel et al. whereby they reported unacceptable high variance in PBMC *GAPDH* expression from 146 T2DM females in Vienna, Austria, which led to *GAPDH* being excluded as a reference gene in that study^51^. Previous studies report that sex is a critical factor in insulin action and associated metabolic pathways and therefore, in susceptibility to developing insulin resistance^52^. This could partially explain the higher variance in PBMC *GAPDH* expression that we observed in T2DM versus NGT females. Further, Bongen et al. have recently reported 144 genes as differentially expressed in PBMCs or whole blood between human males and females^53^. Considering such extensive differences in blood transcriptome between human males and females, in-depth prospective studies on incidence and also on progression of T2DM separately in males and females will pave the way for at least sex-specific, if not, personalized approaches in prevention and management of T2DM.

The strengths of our study include availability of detailed anthopometric, biochemical and clinical data from the subjects that formed part of this study and in-depth analysis of expression stability of genes that are routinely used as reference genes for qRT-PCR based measurements of mRNA abundance in PBMCs. Additionally, unique to this study, we observed lower relative expression of *GAPDH* in female T2DM PBMCs compared to those in female NGTs. This observation might have future implications in understanding sex-specific effects of T2DM on the immune system. A limitation of the current study is the number of potential reference genes that we could analyse in our study samples, due to restricted availability of samples. Few other reference genes *UBE2D2, RPS18*, and *HPRT1*, have not been included in this study as their expression had been found to be stable in human PBMCs under conditions not similar to this study. *UBE2D2* and *RPS18* were reported to be stable in PBMCs collected 4 weeks after influenza vaccination^54^. *HPRT1* was found to be stably expressed in PBMCs of asthmatic patients^55^. It was also found to be stably expressed in PBMC cultures of pulmonary tuberculosis patients^56^.

To conclude, we recommend the use of *YWHAZ* and *ACTB* as reference genes in PBMC-based studies from settings similar to ours. We also report that expression of *GAPDH* and *PPIB* in PBMCs is affected by hyperglycaemia in T2DM patients making them unsuitable as references genes for measuring mRNA expression. Finally, we observed a significant reduction in relative expression of *GAPDH* in female T2DM patients. Lack of existing literature to understand the causes and consequences of female-specific reduction in *GAPDH* expression in PBMCs underlines the need for future, in-depth sex-specific mechanistic studies on T2DM in human subjects.

## Methods

### Study population

This is a retrospective study whereby archived PBMC samples and subject data collected as part of a larger previous study have been used. Patients and samples for this retrospective study were selected from the larger study in which normoglycemic controls (NGT) and type-2 diabetics (T2DM) had been recruited at the Molecular Physiology Lab, Division of Nutrition, St. John’s Research Institute, Bangalore. The subjects had been recruited between 2016 to 2021 from the community in and around St. John’s Research Institute, Bangalore.

The subjects for this retrospective study were selected from the larger study based on the following criteria: (1) Case group of T2DM individuals (n = 39; 20 males, 19 females (T2DM status according to the American Diabetes Association (ADA) criteria^57^ for T2DM: Fasting Blood Glucose > 125 mg/dL and/or HbA1c > 6.5% and/or being clinically managed for T2DM using oral hypoglycaemic agents such as metformin) and (2) Control group of NGTs (n = 47; 29 males, 18 females; availability of sufficient PBMC samples leading to appropriate quality and quantity of RNA extracted from the PBMC samples for carrying out gene expression assays.

### Ethical approval

The study protocol had been approved by the Institutional Ethics Committee of St. John's Medical College and Hospital, Bangalore. The study protocol was explained in the local language of the participants and their signed, informed consent was obtained at the time of recruitment. The study protocol was carried out in accordance with relevant guidelines and regulations.

### Socio-demographics, anthropometry and clinical chemistry

Detailed socio-demographic data for each subject had been collected using a structured questionnaire during subject recruitment for the previous study, from which the samples for this retrospective study were chosen. The individuals had been given the questionnaire in a language that they were most familiar with, and their responses had been recorded in English by competent personnel. Dual-energy X-ray absorptiometry (DXA; DPXMD 7254, Lunar Corporation, Madison, WI) had been used to examine anthropometric parameters for body composition. Weight and height measurements had been taken with 0.1 kg and 0.1 cm precision, respectively. During the recruitment process, each subject had a comprehensive clinical chemistry examination that included standard plasma and serum clinical chemistry assays on fasted blood samples, as described previously^58^.

### Collection of PBMCs

During the recruitment phase for the previous study, 10 mL of blood had been collected from fasting subjects. The blood had been separated into buffy coat (containing PBMCs) and serum/plasma had been utilized for biochemical assays after it was collected. Separated buffy coats had been stored at − 80 °C. These frozen buffy coat samples have been used for the gene expression assays.

### RNA extraction, quality checks and cDNA preparation

RNA was extracted from frozen buffy coats using Trizol reagent (T9424; Sigma-Aldrich, St. Louis, MO, USA), followed by phase separation using chloroform. In brief, total RNA was isolated from the aqueous phase and precipitated with an equal volume of isopropanol. The resulting RNA pellet was washed in 75% ethanol, air-dried, and solubilized in diethylpyrocarbonate treated water. The total RNA obtained was checked for its purity using the Take3 Micro-Volume Plate reader (Synergy H1 Hybrid Multi-Mode Reader, BioTek Instruments Inc, Winooski, USA). Extracted RNA samples with 260/280 and 260/230 ratios of 1.8–2.2 and ≥ 1.8, respectively, were considered for further processing^59,60^.

Total RNA was quantified using Ribogreen reagent (Quant-iT Ribogreen RNA Assay kit, Invitrogen, Eugene, USA) on a fluorescent microplate reader (Synergy H1 microplate reader, BioTek Instruments Inc, Winooski, USA). This was followed by DNAse treatment of the extracted RNA using DNase I kit (Sigma Aldrich, St. Louis, USA). The DNAse-treated samples were reverse transcribed to obtain cDNA using High-Capacity cDNA Reverse Transcription Kit (Applied Biosystems, Luthiana) following the kit manufacturer’s instructions. The obtained cDNA was used for the estimation of transcript abundance using qRT-PCR.

### qRT-PCR assays

Gene expression of *ACTB, PPIB, YWHAZ* and *GAPDH* were assessed in PBMCs from T2DM patients and NGT subjects. Primer sequences were designed for *ACTB, PPIB, YWHAZ* and *GAPDH* qRT-PCR assays (Supplementary Table 3). qRT-PCR reactions were set up in duplicates of 10 µl reactions. The PCR cycling conditions were: 95 °C for 10 min followed by 40 cycles of 95 °C for 15 s, 60 °C for 1 min. All qRT-PCR reactions were performed using SYBR Green master mix (Power SYBR Green PCR Master Mix, Thermo Fisher Scientific, USA) on a QuantStudio 6 Flex Real-Time PCR system (Thermo Fisher Scientific, USA).

Standard curves for the qRT-PCR assays were constructed using cDNA derived from the Universal Human Reference RNA (Agilent Technologies, Santa Clara, CA, USA). The PCR efficiency and correlation coefficients for linearity of the qRT-PCR assays for each housekeeping-gene ranged from 90.027–98.290% to 0.989–0.999, respectively (Supplementary Fig. S1).

Calculation of the relative expression of *GAPDH* and *PPIB* as target genes was done based on a modification of the ΔCt method. The ΔCt was calculated by subtracting the arithmetic mean of the Ct values of the stable reference genes (*ACTB* and *YWHAZ)* from the Ct value of the target gene (*GAPDH/PPIB*) i.e. [ΔCt = Ct_(target gene)_−Ct_(Reference genes mean)_]^61^. The ΔCt values were converted to relative normalization units (RNU = 15—ΔCt) according to Mukhopadhyay et al^43^.

### Assessment of stability of reference genes

For ranking the stability of expression of *ACTB, PPIB, YWHAZ* and *GAPDH* in PBMC samples, the raw mean C_t_ (cycle threshold) values for the qRT-PCR assays were imported to RefFinder^36^, which is a web-based comprehensive tool that integrates the currently available computational programs (GeNorm, Normfinder, BestKeeper, and the comparative Delta-Ct method) to compare and rank the tested candidate reference genes. Based on the rankings from each program, RefFinder assigns an appropriate stability value to an individual gene and calculates the geometric mean of their values for the overall final ranking of the reference genes to determine its stability^36^.

### Statistical analysis

The anthropometric and clinical baseline characteristics of the study subjects were represented as mean ± SD. Normality of distributions were examined using the Shapiro–Wilk test and Q–Q plots. Student’s t-test (for normally distributed data) or Mann Whitney U test (for non-normally distributed data) were utilized to compare the basal parameters, mean Ct values of the reference genes and relative transcript abundances of *GAPDH* and *PPIB* between the study groups. All statistical analyses were done using (Microsoft excel, 2016, Microsoft Corporation, Redmond, Washington, USA) and GraphPad Prism (Version 8, GraphPad Software, San Diego, CA, USA).

## Supplementary Information

Supplementary Information.

## Data Availability

The datasets generated and analysed during the current study that support the findings of this study are available from the corresponding author on reasonable request.
