# Supplementary material for: Identification of stable reference genes in peripheral blood mononuclear cells from type 2 diabetes mellitus patients
Source: Sci Rep. 2023 Jan 10;13:486. doi: 10.1038/s41598-023-27460-3 (PMC9831022; doi:10.1038/s41598-023-27460-3)
Supplement: Supplementary file 1 — Supplementary Information. [file 41598_2023_27460_MOESM1_ESM.pdf]

Supplementary Information

Identification of stable reference genes in peripheral blood mononuclear cells from type 2 diabetes mellitus patients

Ankita Hazarika[a], Bajanai Nongkhlaw[a, b], Arpita Mukhopadhyay[a*]

[a] Division of Nutrition, St. John's Research Institute, St. John's National Academy of Health Sciences, Bangalore, India;
[b]Current address: Department of Pathology, North Eastern Indira Gandhi Regional Institute of Health and Medical Sciences, Shillong, Meghalaya.
*Corresponding author:
Arpita Mukhopadhyay, Ph.D.
Division of Nutrition,
St. John's Research Institute,
St. John's National Academy of Health Sciences,
Sarjapur Road, Bangalore, India.
PIN: 560034
Phone: +91-80-49467000
Email: arpitam@sjri.res.in

Supplementary Table 1. Raw Ct mean values for the 4 reference Genes (*ACTB, GAPDH, YWHAZ, PPIB*).

Subject ID	Gender	Study Group	Ct Mean for *ACTB*	Ct Mean for *GAPDH*	Ct Mean for *YWHAZ*	Ct Mean for *PPIB*
I005	M	NGT	21.9	28.4	26.2	29.4
I008	F	NGT	17.6	22.9	21.5	23.5
I014	F	NGT	17.8	24.6	22.6	25.3
I015	F	NGT	22.5	32.0	29.4	40.0
I018	M	NGT	25.4	31.0	29.4	40.0
I021	F	NGT	18.5	25.7	23.6	26.6
I023	F	T2DM	24.5	40.0	29.1	33.4
I030	M	NGT	21.7	31.6	28.1	31.3
I031	F	NGT	18.3	24.3	22.5	25.0
I032	F	NGT	17.2	23.3	21.4	23.8
I033	M	NGT	21.5	30.2	27.7	30.1
I034	M	NGT	20.2	33.5	26.0	29.1
I035	M	NGT	18.5	29.6	23.5	26.5
I041	M	NGT	24.0	36.7	28.6	32.3
I043	F	T2DM	21.7	32.0	27.2	29.5
I048	M	NGT	19.7	29.9	24.7	28.4
I051	M	NGT	20.5	27.4	23.2	26.0
I053	M	NGT	20.4	33.2	25.6	28.7
I055	M	NGT	19.6	29.7	24.8	28.9
I058	M	NGT	25.1	33.7	29.4	32.0
I059	F	NGT	20.6	29.5	26.5	29.0
I060	M	NGT	21.8	32.6	25.9	30.6
I061	M	NGT	25.6	34.3	30.4	40.0
I064	M	NGT	24.7	32.5	29.2	40.0
I067	M	T2DM	23.1	31.6	29.3	33.0
I072	M	T2DM	24.9	31.9	28.5	32.8
I074	M	T2DM	21.8	30.3	27.7	32.0
I075	M	T2DM	22.1	35.3	28.3	32.9
I081	M	T2DM	24.3	32.7	29.4	33.1
I083	F	NGT	23.5	40.0	40.0	40.0
I085	M	T2DM	22.4	30.4	27.9	31.4
I089	M	T2DM	17.1	23.6	21.4	24.3
I095	M	T2DM	24.5	35.2	29.1	31.8
I097	F	NGT	19.0	26.0	22.8	25.8
I101	F	T2DM	21.6	32.0	27.3	30.6
I107	F	T2DM	18.2	26.7	23.4	26.7
I109	F	T2DM	24.9	32.8	28.9	40.0
I110	M	T2DM	25.9	40.0	32.3	40.0
I113	M	T2DM	20.2	27.5	24.8	26.9
I116	M	NGT	23.6	28.8	28.4	31.2
I119	M	T2DM	19.9	27.3	23.7	27.9
I120	F	T2DM	21.6	30.0	26.7	30.0

I121	M	T2DM	22.7	31.1	27.4	31.3
I129	F	T2DM	22.6	30.4	27.9	31.4
I139	M	T2DM	23.7	31.9	31.4	31.0
I140	F	NGT	20.7	27.9	25.2	27.5
I145	M	NGT	18.8	25.3	24.4	25.5
I147	M	NGT	26.4	40.0	31.4	40.0
I153	M	NGT	21.3	29.5	27.5	29.1
I154	F	NGT	18.8	25.6	24.2	25.0
I156	M	NGT	22.7	31.9	27.6	31.1
I166	M	NGT	18.5	24.3	23.9	25.4
I170	M	NGT	24.5	33.4	29.9	32.9
I172	M	NGT	25.5	32.7	32.1	32.6
I178	M	NGT	22.6	30.6	29.3	40.0
I180	F	NGT	19.3	26.6	24.4	27.4
I181	M	T2DM	23.6	30.1	28.4	31.9
I184	F	NGT	25.9	33.8	30.0	40.0
I191	F	T2DM	20.5	26.7	23.2	26.7
I196	F	NGT	18.6	23.9	22.4	24.2
I199	F	T2DM	21.3	30.7	26.9	29.7
I204	M	NGT	20.1	25.5	28.5	26.8
I205	F	NGT	23.0	31.9	23.5	32.7
I206	F	T2DM	21.9	33.8	28.5	33.1
I207	F	T2DM	18.4	25.2	22.2	24.8
I210	M	NGT	20.3	29.1	23.7	29.2
I211	F	T2DM	20.5	28.8	25.9	29.1
I212	M	T2DM	18.2	25.6	22.5	25.2
I217	M	T2DM	23.4	40.0	29.4	33.9
I218	F	T2DM	17.1	23.0	20.4	22.6
I221	M	T2DM	25.8	40.0	40.0	32.7
I222	M	T2DM	20.7	26.4	25.4	28.1
I223	F	T2DM	18.1	27.6	22.6	27.0
I224	F	T2DM	18.2	25.6	22.7	27.3
I226	M	NGT	19.2	29.8	25.6	29.9
I227	M	NGT	16.9	24.4	23.0	25.8
I229	M	T2DM	22.7	30.9	28.3	34.7
I231	F	T2DM	25.5	31.0	28.1	33.5
I233	F	T2DM	17.3	25.4	21.2	24.7
I234	F	T2DM	21.4	29.9	25.6	30.9
I235	F	NGT	17.5	29.0	23.4	26.5
I236	F	T2DM	20.6	29.7	25.7	29.6
I238	M	NGT	18.5	28.2	23.7	26.6
I239	F	NGT	18.8	25.0	23.6	26.0
I240	M	T2DM	19.6	27.5	24.8	29.2
I241	F	NGT	19.5	27.5	24.0	27.4

Supplementary Table 2. Relative expression of *GAPDH* and *PPIB* (expressed as Relative Normalization Unit, RNU)[43] using *ACTB* and *YWHAZ* as reference genes in the 86 study subjects. *P* values are from Mann–Whitney test.

**Reference genes**		**All (n=86)**	**NGT (n=47)**	**T2DM (n=39)**	***P***	**NGT Males (n=29)**	**T2DM Males (n=20)**	***P***	**NGT Females (n=18)**	**T2DM Females (n=19)**	***P***
GAPDH	Mean ± SD	8.6 ± 2.9	8.9 ± 2.8	8.3 ± 3.1	0.372	8.6 ± 2.8	8.0 ± 3.8	0.789	9.3 ± 2.8	8.5 ± 2.4	**0.034**
	Median (Q1, Q3)	9.4 (7.8, 10.4)	9.4 (7.7, 10.8)	9.4 (8.1, 9.9)		8.8 (7.5, 10.6)	9.6 (8.4, 9.8)		10.1 (9.1, 11.0)	9.0 (8.1, 9.9)	
	Range (Min, Max)	13.7 (0.0, 13.7)	13.7 (0.0, 13.7)	11.7 (0.0, 11.7)		13.7 (0.0, 13.7)	11.7 (0.0, 11.7)		11.7 (0.0, 11.7)	10.9 (0, 10.9)	
PPIB	Mean ± SD	8.3 ± 3.3	8.0 ± 3.8	8.6 ± 2.5	0.271	7.9 ± 3.8	8.7 ± 2.8	0.758	8.2 ± 4.0	8.5 ± 2.3	0.113
	Median (Q1, Q3)	9.1 (8.2, 10.1)	9.4 (8.2, 10.3)	8.8 (8.2, 10.0)		9.1 (8.2, 9.8)	8.8 (7.9, 10.0)		9.7 (9.0, 10.4)	8.8 (8.3, 9.5)	
	Range (Min, Max)	15.2 (0.0, 15.2)	12.5 (0.0, 12.5)	15.2 (0.0, 15.2)		12.5 (0.0, 12.5)	15.2 (0.0, 15.2)		11.5 (0.0, 11.5)	11.1 (0.0, 11.1)	

Supplementary Table 3. Primer sequences and amplification efficiencies of qRT-PCR assays for the 4 reference genes.

Gene Name	Primer	Prime sequence (5’ to 3’)	Primer efficiency (%)	Coefficient for linearity (R^2)
ACTB	Forward	TTCCTGGGCATGGAGTC	90.027	0.999
	Reverse	CAGGTCTTTGCGGATGTC		
GAPDH	Forward	CTCTGACTTCAACAGCGA	91.714	0.989
	Reverse	TTCGTTGTCATACCAGGA		
PPIB	Forward	GCCAACGCAGGCAAAGACAC	92.615	0.999
	Reverse	GCACCACCTCCATGCCCTCTA		
YWHAZ	Forward	ACTTTTGGTACATTGTGGCTTCAA	98.290	0.996
	Reverse	CCGCCAGGACAAACCAGTAT		

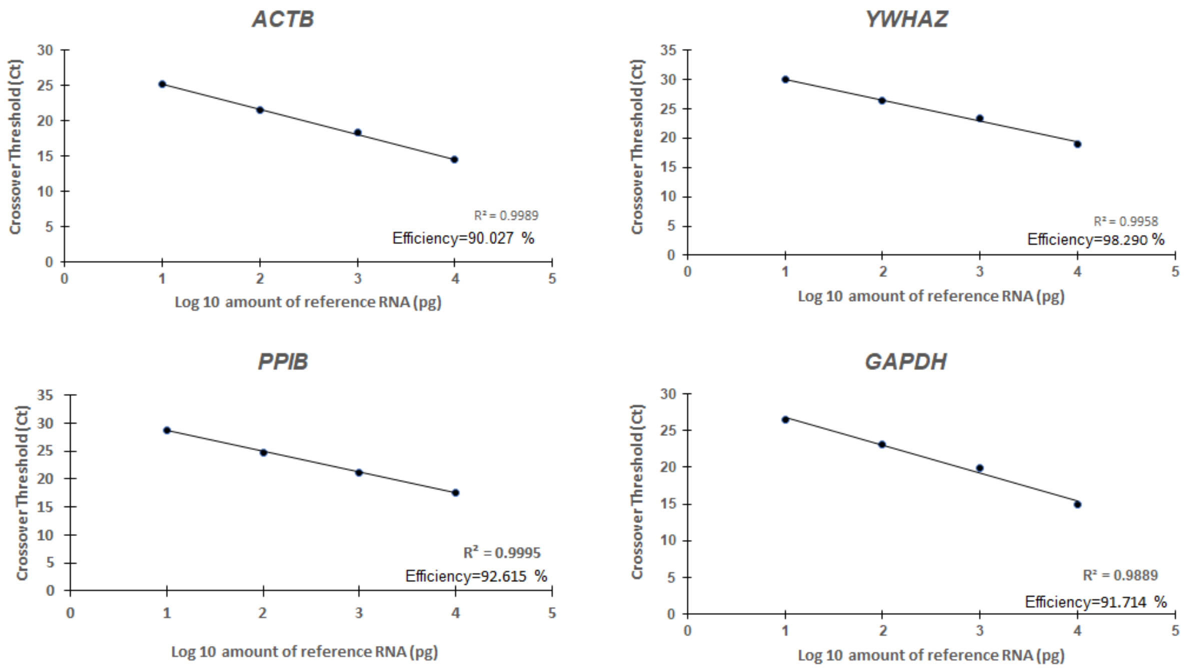

Supplementary Figure S1. Standard curve of the 4 reference genes (*ACTB, GAPDH, YWHAZ* and *PPIB*) in qRT-PCR assay using cDNA derived from human universal RNA as PCR template.
